# Tissue Engineering and Regenerative Medicine in Pediatric Urology: Urethral and Urinary Bladder Reconstruction

**DOI:** 10.3390/ijms23126360

**Published:** 2022-06-07

**Authors:** Martina Casarin, Alessandro Morlacco, Fabrizio Dal Moro

**Affiliations:** Department of Surgery, Oncology and Gastroenterology, Giustiniani 2, 35128 Padua, Italy; martina.casarin@unipd.it (M.C.); fabrizio.dalmoro@unipd.it (F.D.M.)

**Keywords:** biomaterial, tissue engineering, urinary bladder, urethra, regenerative medicine, hypospadias, neurogenic bladder, bladder exstrophy, pediatric urology

## Abstract

In the case of pediatric urology there are several congenital conditions, such as hypospadias and neurogenic bladder, which affect, respectively, the urethra and the urinary bladder. In fact, the gold standard consists of a urethroplasty procedure in the case of urethral malformations and enterocystoplasty in the case of urinary bladder disorders. However, both surgical procedures are associated with severe complications, such as fistulas, urethral strictures, and dehiscence of the repair or recurrence of chordee in the case of urethroplasty, and metabolic disturbances, stone formation, urine leakage, and chronic infections in the case of enterocystoplasty. With the aim of overcoming the issue related to the lack of sufficient and appropriate autologous tissue, increasing attention has been focused on tissue engineering. In this review, both the urethral and the urinary bladder reconstruction strategies were summarized, focusing on pediatric applications and evaluating all the biomaterials tested in both animal models and patients. Particular attention was paid to the capability for tissue regeneration in dependence on the eventual presence of seeded cell and growth factor combinations in several types of scaffolds. Moreover, the main critical features needed for urinary tissue engineering have been highlighted and specifically focused on for pediatric application.

## 1. Introduction

Several congenital urological diseases, including posterior urethral valves, bladder exstrophy, and neurogenic bladder [1], can involve pediatric patients and require reconstruction or replacement of dysfunctional genitourinary tissues and organs. Normal urethra formation can be affected by hypospadias, a congenital disorder characterized by the interruption of normal urethral development and an abnormal location of the meatus, a curvature of the penis (chordee), and an incomplete ventral prepuce [2,3]. Moreover, congenital disorders can affect the urinary bladder through bladder exstrophy and neurogenic bladder, which result in reduced organ capacity, impaired compliance, incontinence, and possibly renal damage [1].

The current gold standards provide a urethroplasty procedure in the case of urethral malformations and bladder reconstruction, which often involves enterocystoplasty, in the case of urinary bladder disorders. However, both surgical procedures are associated with severe complications, including fistulas, urethral strictures, and dehiscence of the repair or recurrence of chordee in the case of urethrocystoplasty [2] and intestinal obstruction, metabolic disturbances, stone formation, urine leakage, and chronic infections in the case of enterocystoplasty [1].

As the main issue involves the lack of sufficient and appropriate autologous tissue, tissue engineering (TE) is currently considered a possible solution. TE is the combination of biomaterials and bioengineering principles with cell implantation or the directed growth of host cells to develop a tissue or an organ that can substitute native tissue, both in structure and function [4]. Thus, the goal of regenerative medicine is to restore the function of tissues and organs by developing a functional substitute that resembles native tissue in biological and mechanical properties, with a minimal or an absent immunological response [5].

Hitherto, several TE approaches have been evaluated, starting from the choice of scaffold and followed by the choice of cell type in the case of seeded grafts [6]. In addition to the requirement of biocompatibility, biodegradability, mechanical features similar to native tissue, assistance on cell adhesion, and surgically easy manipulation for the choice of the ideal scaffold, in the case of urinary applications further requirements are crucial, including quick regeneration of the urinary barrier to minimize leaks that would heighten the local inflammatory response and appropriate mechanical resistance to sustain the mechanical forces necessary for bladder filling and emptying [2,6,7,8,9]. Moreover, in the specific case of pediatric urology, a greater regenerative capacity of the scaffold, a long life span, and no requirement for prolonged urinary diversions with catheters are crucial elements [1,3].

In the light of these requirements, various strategies have been studied in order to find the most appropriate scaffold, including natural polymers (e.g., polymers derived from collagen [10,11,12,13,14], chitosan, gelatin, alginate [15], or silk [16,17,18,19,20]); natural scaffolds derived from decellularized tissues (e.g., small intestinal submucosa (SIS) [21,22,23,24,25], bladder acellular matrix (BAM) [15,26,27], amniotic membrane (AM) [28], and dermis [29]); synthetic scaffolds (e.g., poly(glycolic acid), poly(lactic acid), and poly(lactic-co-glycolic acid) [30,31,32,33,34]); and hybrid scaffolds that are generated by using a combination of synthetic and natural materials [35,36,37,38,39] (e.g., coupling of BAM with PLGA [39], BAM with PGA [40], AM with PLCL [38], and PLCL with collagen [36,41]).

Despite the promising solution that TE could offer to pediatric surgical applications, the variable degree of success and the possible complications, such as fistulae or strictures in the case of urethral reconstruction and early tissue fibrosis, lack of vascularization, insufficient urine barrier, and inadequate contractility in the case of urinary bladder, represent the major challenges to transferring this approach into clinical routine [3,42,43].

The present review will focus on urethral tissue-engineering methodologies to be addressed for hypospadias cases and for urinary bladder reconstruction in the case of pediatric disorders.

## 2. Requirements for Tissue Engineering in the Case of Pediatric Urology

The actual gold standards in the case of urinary congenital malformations need the use of autologous tissues for urinary reconstructions. In the case of severe hypospadias, extra tissue is frequently required to restore the missing urethra. Autologous sources for urethral replacement usually involve skin from the genital areas or extragenital regions [44,45,46]. In fact, the most widely used replacements are preputial skin graft/flaps when available, or buccal mucosa (BM) free grafts [47,48], when considered appropriate. However, the several aforementioned complications associated with this surgical procedure often lead to multiple surgeries for each patient [30,49,50].

For these reasons, recent efforts have been focused on TE in order to spare autologous tissues, while preventing invasive multiple surgeries and potential complications.

The first aim of TE is to provide the ideal scaffold, which has to be biocompatible, biodegradable, and non-immunogenic, and it has to promote adequate blood supply, enhance cell adhesion and native-like tissue organization, and provide a substrate with similar mechanical features to those of native tissue. Specifically, with regard to urological applications, the scaffold has to provide an effective barrier against cytotoxic urine for the surrounding tissues and has to guarantee adequate mechanical features in order to resist to the cyclical filling and emptying of the urinary bladder [1,3,6]. Engineered bladders have to support urine storage while keeping contractile properties to allow physiologic voiding, and they need to reconstruct a compliant muscular wall with a highly specialized urothelium [7]. Therefore, the ideal artificial bladder should possess properties like those of the native urinary bladder; it should possess the ability to store urine at low pressure and should allow voluntary voiding with minimal reflux. However, while urothelium demonstrated the ability to regenerate generously over free grafts, bladder muscle tissue is less likely to regenerate in a normal fashion. For this reason, it was hypothesized that building a 3D bladder construct in vitro before implantation would facilitate the differentiation of cells after implantation in vivo and would minimize the inflammatory response toward the matrix, avoiding graft contracture and shrinkage [51].

Bladder replacements should provide mechanical support and adequately endure the forces exerted by the surrounding tissues. Moreover, the biomaterials used for bladder reconstruction should be easily manipulated into a hollow, spherical configuration [52]. The presence of cells could be necessary to obtain the adequate tissue structure and function of the reconstructed bladder. In fact, cells implanted on the scaffold have the function of strengthening the mechanical properties of the scaffold, providing an impermeable barrier to urine, and stimulating the scaffold remodeling, while secreting trophic factors enhancing the regeneration process [53].

In the case of pediatric issues, further requirements are imperative, including a good growth potential of the scaffold, a long life span, and no prolonged urinary diversions with catheters [1,3].

The initial TE approaches were focused on biomaterial choice with the aim of providing a sufficient structural support and an adequate environment to promote cellular growth. However, the limitations of the regenerative potential of simple scaffolds led to the necessity of cell involvement by pre-seeding the scaffolds in vitro. For this reason, the choice of cell type became as crucial as the choice of appropriate scaffold type. 

The primary cell source is autologous donor cells, which have to be expanded in vitro and then seeded on the appropriate scaffold and finally implanted into the specific body site. Autologous cells should avoid the risk of rejection and the associated complications [54,55]. For example, in the case of urinary bladder reconstruction or substitution, urothelial cells (UCs) and smooth muscle cells (SMCs) are generally required to obtain a urinary barrier and to provide an adequate structural support. However, in some cases, such as neuropathic patients, the UCs and SMCs did not have the same functional and regenerative potential as normal cells [56,57]. In fact, smooth muscle cells isolated from neuropathic bladders have shown abnormal growth, less contractile ability, and inferior adherence compared to normal cells [56]. Moreover, the genetic profile in neuropathic bladder smooth muscle cells was found to be altered. For these reasons, the SMCs derived from diseased bladders may not be appropriate for tissue-engineering purposes. Similarly, autologous UCs derived from patients with interstitial cystitis or another form of chronic cystitis or neuropathic bladder, posterior urethral valves, epispadias, and non-neurogenic bladder dysfunction have a reduced capacity for in vitro proliferation and differentiation [57,58,59].

For these reasons, alternative cell sources were found in buccal mucosa [60,61], in order to provide a barrier function for the urothelium layer, or in stem cells, in order to regenerate the smooth muscle and urothelium layers [62].

In fact, stem cells offer great promise when autologous cells cannot be used. The commonly available stem cells include embryonic stem cells (derived from human embryos and aborted fetuses); adult stem cells (derived from tissues that develop from the three embryonic germ layers); umbilical cord blood stem cells (which are multipotent stem cells similar to adult stem cells); amniotic fluid stem cells (which are pluripotent and can differentiate into all three germ layers with low immunogenicity and high anti-inflammatory action); placental stem cells (which are multipotent adult stem cells); induced pluripotent stem cells (which are derived from the patient’s tissue and induced into pluripotency); and, finally, urine-derived stem cells (which are derived from parietal cells or podocytes within glomerulus in the kidney and can be isolated form voided urine [63]) [64]. In the case of the bladder, there are two commonly employed techniques for using stem cells: implantation of the stem cells in vivo with pre-differentiation or induction of the stem cell differentiation toward the specific target cells in vitro, followed by implantation in vivo [65]. Moreover, several studies demonstrated worse results in the case of unseeded scaffolds for urinary bladder reconstruction as they led to fibrosis and shrinkage, while the seeding of cells could prevent this complication.

In order to allow cells to survive and proliferate on the scaffold, they need specific metabolic and nutritional conditions, which are achieved by using specific bioreactors [66]. In the specific case of smooth muscle cells, whose regeneration is more difficult in comparison with the urothelial ones, partial or complete regeneration was observed in the cell-seeded scaffolds; however, their morphology and function were not completely equivalent to those of native bladder smooth muscles [67]. For this reason, the application of an electrical stimulation in a cell culture in vitro could induce a proper smooth muscle fiber arrangement and function in vivo.

Another challenging issue of functional bladder regeneration is innervation as the storage in the urinary bladder depends on the autonomic nervous system. Luckily, it was demonstrated that Schwann cell seeding or the application of exogenous neurotrophic factors could induce bladder innervation [68,69].

## 3. Tissue-Engineered Urethra in the Case of Hypospadias

Hypospadias is one of the most frequent and complex genitourinary congenital malformations encountered in children. It occurs in approximately 0.2 to 4.1 in 100 live births [70,71]. The prevalence in Europe is 19.9 in 10,000, in the USA it is 34.2 in 10,000, in South America it is 5.2 in 10,000, in Asia it is 0.6–69.0 in 10,000, in Africa it is 5.9 in 10,000, and in Australia it is 17.1–34.8 in 10,000 [72].

Hypospadias results from the malposition of the urinary meatus on the ventral aspect of the penis following incomplete closure of the urethral folds [2]. The specific factors that contribute to the development of this malformation remain elusive and are likely a combination of genetic and environmental components [73]. In particular, the environmental factors which potentially contribute are endocrine disruptors and those causing epigenetic modifications which alter gene expression. Pesticides, exogenous hormones, phthalates, and phytoestrogens are all suspected of potentially altering the gestational hormones milieu [74,75].

Many are the challenges that the surgeon has to face in order to obtain functionality and cosmetically satisfactory results [76]. The treatment of hypospadias is surgical and involves the repair of the urethral defect and the correction of the ventral curvature. There is no consensus about the best approach, and the choice depends on patient anatomy and surgeon preference [77]. However, there are several associated complications, which include fistulas, urethral strictures, dehiscence of the repair, and the recurrence of chordee. In particular, the outcome of the urethroplasty depends on the quality of the anatomical structures and the surgical approach, but it is also dependent on the availability of the appropriate graft source as patients with severe hypospadias frequently need extra tissue to restore the missing urethra [2]. Usually, the autologous graft sources used for urethral replacement are skin from the genital areas or extragenital regions [44,45,46] or, more recently, buccal mucosa free skin [47], because of its easier harvesting procedure, which causes minimal discomfort for the patient, and because of its acceptable degree of morbidity [78]. 

The disappointingly low success rates, the complications related to graft harvesting, and the tendency toward graft deterioration over time led the attention to TE to tailor grafts with features like those of the native urethra, which can always be available [2]. In the case of hypospadias, the creation of a long, tubular construct with the ability to facilitate angiogenesis and fast regeneration is required. 

Several approaches have been investigated up until now; however, the ideal solution has not been found yet as strictures and fistula formation, due to low vascularization, prolonged inflammation, and fibrosis, have been observed in most studies.

In 1999, Atala et al. [47] performed hypospadias repair by using a collagen-based matrix obtained from cadaver bladder submucosa on four patients (aged 4–20 years old) who had undergone repeated hypospadias surgical repair. The neourethra length ranged from 5 to 15 cm, and the 22-month follow-up revealed a successful outcome in three of the four patients with regard to cosmesis and function.

Similar results were also obtained by El Kassaby et al. [79], who used the same type of matrix in 28 patients (aged 22–61 years old) who suffered from urethral stricture. The length of the neourethra ranged from 1.5 to 16 cm. Voiding patterns, physical examination, retrograde urethrography, uroflowmetry, and cystoscopic examinations were performed preoperatively and postoperatively, and urethral biopsies were performed, revealing a successful outcome after a 36–38-month follow-up in 24 of the 28 patients. The remaining four patients had a slight caliber decrease at the anastomotic sites, and a subcoronal fistula developed in one patient; the fistula closed spontaneously after 1 year. Additionally, adequate caliber conduits and normal urethral tissues have been observed.

Subsequent randomized studies aimed to assess the outcome of bladder acellular matrix (BAM) compared with that of buccal mucosa, which is a gold standard in urethral grafting [80]. Thirty patients (aged 21–59 years old) with strictures were enrolled and underwent treatment for strictures ranging from 2 to 18 cm. The follow-up lasted from 18 to 36 months, showing a 100% success rate in the case of patients treated with buccal mucosal grafts and an 89% success rate in the case of patients treated with BAM; this was in the case of patients with only one or no previous interventions. Instead, in the case of patients with two or more previous operations, the success rate achieved in the case of the buccal mucosa graft-treated patients was 100%, while in the BAM-treated ones the success rate was only 33.3%, showing how the results mostly depend on the quality of the urethral mucosal bed and the ability of the native urethra to regenerate along the scaffold material. Huang et al. [81] evaluated the effect of the reconstruction of the penile urethra with 3D porous BAM in 30 rabbits (15 with peracetic acid-treated BAM and 15 with non-treated BAM), showing enhanced urothelium, smooth muscle regeneration, and neovascularization in the peracetic acid-treated ones. Fu et al. [82] implanted tubular acellular collagen matrices (1.5 × 1 cm^2^) in rabbit models from allogeneic rabbit bladder submucosa, unseeded (nine rabbits, control group) and seeded with foreskin epidermal cells (nine rabbits). Better results were achieved with the seeded scaffolds, where a wide urethral caliber was maintained with no sign of strictures. Moreover, the seeded grafts formed a single-layer structure after 1 month, and at 2 and 6 months, there were several layers of epidermal cells with abundant vessels in the submucosa. Li et al. [83] used BAM obtained from the bladders of rabbits for urethral reconstruction (2.2 × 1.0 cm^2^); this was implanted in 24 male rabbits (12 received unseeded grafts and 12 seeded grafts with oral keratinocytes). All the animals implanted with seeded scaffolds survived until sacrifice: they voided without difficulty, and no signs of discomfort after catheter removal were observed. Moreover, a wide urethral caliber was maintained without stricture, leakage, or dilatation. Conversely, in the control group, 2 of the 12 rabbits died of urinary tract infection, another 2 had fistulas, and the remaining 8 revealed strictures. In the unseeded group, the urethras appeared damaged, stiff, pallid, and affected by strictures. 

In a more recent study, Cao et al. [84] evaluated the effects of urethral regeneration with pre-vascularized BAM hydrogel (BAMH)/silk fibroin (SF) composite scaffolds in 30 rabbits which were first incubated in the omentum for 2 weeks in order to increase neovascularization. Once implanted to repair autologous urethral defects, the scaffolds resulted in the regeneration of the urethral epithelium and the smooth muscle, demonstrating the potential for urethral reconstruction.

Another biological tissue investigated for urethral repair was SIS, which was widely reported for its capability to integrate with host tissues and contribute to tissue regeneration, providing an excellent microenvironment for cell adhesion and proliferation, while simultaneously promoting tissue repair [85]. Le Roux [86] evaluated the use of SIS as a substitute for skin in endoscopic urethroplasty in nine patients with bulbar strictures. However, the study was unsuccessful. Unsuccessful results were also obtained in the case of stricture repair using four-layer SIS [87] in five patients (aged 61–68 years old) with recurrent urethral strictures (3.5–10 cm length); in four patients, the operation was not successful, perhaps due to the lack of acellularity of the scaffold used [88]. In shorter strictures (4–10 cm length) in 20 patients (20–74 years old), the use of SIS was associated with an 85% success rate with a follow-up of 13–35 months [89]. A similar rate of success (80%) was achieved in 50 patients (45–73 years old) with anterior urethral stricture by using four-layered SIS patches [90]. In another study, Orabi et al. [91] used four-layer SIS in 12 patients (1.5–15 years old) with hypospadias. Nine patients voided normally with a good cosmetic appearance and no postvoid residual urine. Six patients had a successful repair with no further intervention, and three had small fistulae, while in three patients the graft failed, by complete disruption or stricture. Moreover, graft infection occurred in three patients. More recently, SIS was used for the repair of a large urethral defect in 22 male rabbits, both unseeded and seeded with UCs harvested noninvasively by washing the urinary bladder with saline solution [92]. The authors found that cell-seeded transplants were superior to unseeded ones in terms of the regeneration of a stratified epithelium similar to a native urethra.

Another tissue tested for urethral reconstruction was derma. Fossum et al. [93] used acellular dermal matrix seeded with bladder urothelium cells obtained by lavage in six patients (aged 14–44 months) with severe hypospadias. After a follow-up of 3–5.5 years, all the patients could void thorough their neourethra without straining and with no residual urine after micturition. Only one patient developed stricture, which was treated conservatively with persisting good effect. Two patients developed a fistula which required surgical correction, and the last one developed an obstruction in the proximal anastomosis, which was treated with an internal urethrotomy. The same author [94] evaluated the long-term effects (6–8 years) on hypospadias repair with acellular dermis seeded with autologous urothelial cells harvested by bladder washing. The outcome was assessed with respect to cosmetic appearance, voiding function, urinary flow, artificial erection, urethroscopy, and biopsies. All the patients presented with a good cosmetic appearance, and the urinary flow curves were bell-shaped in all but one. However, the limitation of this study was the small group of patients and the lack of controls. Morgante et al. [95] compared two acellular tissue matrices: a full-thickness acellular bladder matrix (PABM) and a commercially sourced cross-linked matrix from porcine dermis (Permacol). After implantation in 12 pigs (6 received PABM and 6 received Permacol), they saw the full incorporation of PABM, while the Permacol remained palpable after 3 months. Moreover, the PABM graft region was extensively vascularized and completely infiltrated by cells, while the Permacol remained acellular.

Another biomaterial used for urethral regeneration was amniotic membrane (AM). Shakeri et al. [96] used human amniotic membrane (5 × 10 mm^2^ patch) to repair uroepithelium injuries in 20 healthy rabbits, which were studied for a month for any sign of infection and fistula formation. A complete re-epithelialization was observed on the reconstructed urethra, one case of infection and fistula was noted, and two cases of urethral strictures were reported. In another work, Gunes et al. [97] evaluated and compared the success of AM and BM grafts and their simultaneous use in the case of penile augmentation urethroplasty in 12 rabbits, finding that the group with AM + BM grafts may provide better neovascularization and epithelial transformation with minimal or no subepithelial connective tissue proliferation.

Another type of tissue used for urethral regeneration in 24 male rabbits was preputial acellular matrix (PAM) obtained by the decellularization of prepuces from circumcised boys [98]. The results showed that PAM combined with fibrin sealant may be a reliable option for repairing segmental urethral defects.

Decellularized urethral tissue was also successfully used in order to regenerate urethra. Kajbafzadeh et al. [99] used human decellularized urethra which were first implanted in rat omentum and then located into the scrotum, obtaining better results compared to in vitro seeded decellularized urethra. Another group [100] decellularized porcine urethras and recellularized them with human muscle progenitors cells, human skeletal myoblasts, and adipose-derived stromal vascular fractions, providing a suitable environment for cellular adhesion and proliferation.

Among the various naturally derived scaffolds for urethral regeneration, collagen-based scaffolds have been investigated. De Filippo et al. [101] used tubularized collagen scaffolds obtained from decellularized bladder submucosa, both unseeded and seeded with epithelial cells within the lumen and SMCs on the outer surface. The product was implanted in 24 male rabbits (12 unseeded and 12 seeded). Better results were obtained in the case of the seeded scaffolds, which entailed the regeneration of the normal urethral architecture and the maintenance of a wide urethral caliber without any sign of strictures and biomechanical features similar to those of normal urethra. Pinnagoda et al. [12] engineered a two-layer acellular high-density collagen tube (2 cm long) using rat-tail collagen implanted in 20 male rabbits after subtotal excision of the urethra. The authors saw the spontaneous repopulation of UCs and SMCs on all grafts, with 20% of both fistula and stenosis. In another study [102], tubular collagen scaffolds were designed in order to mimic the dynamics of the human urethra by compressing them on star-shaped mandrel. The scaffolds were then seeded with human epithelial cells and cultured in a bioreactor under dynamic conditions mimicking urination. Mikami et al. [103] fabricated tissue-engineered urethral grafts using autologously harvested oral cells (epithelial cells from mucosa and muscle cells). The muscle cells were seeded on collagen type I mesh matrices obtained from rat tails in order to control cell growth orientation, and then, a stratified epithelial cell sheet was joined together, forming a two-layer graft (2 × 2.5 cm^2^). The grafts were then implanted in 10 male dogs (experimental group) while other 10 dogs (control group) underwent only the urethral operation without grafting. After a follow up of 12 weeks, the experimental group showed the maintenance of a wide urethral caliber without stricture, leakage, or dilatation, while urinary fistula and severe strictures were found in the control group.

Another naturally derived scaffold, which showed better elasticity and relative non-immunogenicity than other biomaterials, including collagen and PLA, is silk fibroin (SF). Chung et al. [20] compared bi-layered SF acellular scaffolds with SIS in rabbits to repair urethra; they demonstrated similar degrees of tissue regeneration but reduced immunogenicity in the case of SF. Another group [104] evaluated a composite scaffold made of human keratin, silk, gelatin, and calcium peroxide (CPO) for urethral repair in dogs, demonstrating improved organized muscle bundles and epithelial layer. Xie et al. [19] investigated the use of tissue-engineered buccal mucosa (TEBM) with SF matrices (5.0 × 1.5 cm^2^ urethral mucosal defect) in 10 female dogs (5 animals received SF matrices as the control and 5 animals received TEBM as the experimental group). In the TEBM group, keratinocytes were seeded in the inner side, while in the outer side fibroblasts were cultured. The animals in the experimental group did not show any sign of stricture and the epithelial cells covered the defect and formed stratified layers at 6 months. The keratinocytes could act as a barrier to protect the underlying tissues from the cytotoxic effects of urine, while in the control group urine permeated the urethral defect causing severe inflammation and extensive fibrosis. 

Moreover, synthetic polymers, which can be non-degradable or biodegradable, have been evaluated for the urinary tract reconstruction. Among the non-degradable ones, which generally do not promote cellular attachment and tissue regeneration and require surface treatment to allow urothelial cell adhesion [105], polytetrafluoroethylene (PTFE) and poly(ethylene terephthalate) have been tested. Anwar et al. [106] used PTFE for urethral replacement in 10 dogs showing the development of fibrous tubes around the graft without evidence of the regeneration of normal urethral tissue, as well as the formation of calcification and fistula at 6 months. Romagnoli et al. [107] used tubular PTFE (Gore-Tex) grafts in eight patients (1.5–14 years old) with proximal hypospadias. However, one developed fistula, which required intervention, and all the patients developed mild stenosis.

Better results were achieved by biodegradable synthetic polymers, such as poly-L-lactide (PLL), polycaprolactone (PCL), and poly(lactic-co-glycolic acid) (PLGA), as the mechanical properties, such as the porosity and the degradation rate can be tailored to satisfy the requirements of urethral reconstructions [2]. Olsen et al. [108] replaced canine urethra (4 cm length) with grafts consisting of polyglactin fiber mesh tube coated with polyhydroxy butyric acid. Almost complete regeneration of the urethral epithelium was achieved, and the neourethra remained patent, and there were no anastomotic strictures or inflammatory reactions around the urethra. Sartoneva et al. [109] compared different poly-L-lactide-co--caprolactone (PLCL)-based membranes: smooth (s) and textured (t) PLCL and knitted PLA mesh with compression-molded PLCL (cPLCL), seeding human UCs. Both sPLCL and tPLCL supported cell growth significantly better than cPLCL. The same author [110] compared the effects of PLCL to acellular human amniotic membrane on human UC viability, proliferation, and differentiation, showing better results in the case of PLCL. Lv et al. [111] evaluated composite scaffolds obtained through the combination of poly(L-lactide) (PLLA)/poly(ethylene glycol) (PEG). The scaffolds seeded with human amniotic mesenchymal cells (hAMSCs) and implanted in rabbits (2 cm long) in the urethras gave better results compared to the unseeded ones.

Raya-Riviera et al. [112] produced tissue-engineered urethras by using tubularized poly(glycolic acid) (PGA): PLGA scaffolds seeded with autologous SMCs and Ucs, which were implanted in five patients (10–14 years old) with urethral defects. The scaffolds remained functional in a clinical setting for up to 6 years, without fistula and urinary infections. More recently, Zhang et al. [113] evaluated a novel drug-delivering nanoyarn scaffold in a dog model in the case of urethroplasty, with the aim of continuously delivering ICG-001 (a Wnt signaling inhibitor which could effectively suppress fibroblast proliferation and fibrotic protein expression) during tissue reconstruction. They compared conjugated nanofibrous scaffold (two dogs), nanoyarn without ICG-001 (four dogs), and nanoyarn treated with ICG-001 (four dogs) by creating ventral urethral defects in the penile part of the dogs and by using 2 cm-long scaffolds. The drug-delivery nanoyarn showed the most effective clinical advantage in treating the urethral defects, and the authors suggested that it could serve as a promising method for curing human patients with urethral defects.

In another recent study by Niu et al. [114], a multifaceted bio-interface nanofiber tissue-engineered tubular scaffold graft was designed and evaluated by using an alternating block polyurethane and hydrophilic PEGylation interface capable of promoting urethral epithelial cells (ECs) and SMC adhesion, directional extension, and proliferation. After 3 months of in vivo implantation in rabbits, the authors observed a local neo-vascularization in the scaffolds with a coating rich in seed-cell matrix, facilitating oriented SMC remodeling and lumen epithelialization as well as patency.

Recently, the fabrication of urethras by using 3D bioprinting technology, which can contain spatially arranged UCs and SMCs, was presented for the first time [115]. The authors used PCL and PLCL for the scaffold component, while the cell-laden hydrogel (bioink) had fibroin, gelatin, and hyaluronic acid as the main components. Bladder UCs and SMCs maintained viability and proliferation in the hydrogel after 7 days.

Until now, several types of scaffolds and cells have been evaluated in order to reconstruct urethra, and various animal models and surgical repairs have also been investigated (in Table 1 and Table 2, the studies performed in animal models and in patients are summarized, respectively). However, the ideal solution has yet to be found. The currently available experience seems to suggest that longer defects need pre-seeded scaffolds, while for the shorter ones this might not be necessary. Even if the ideal scaffold has not already been found, a possible solution could be the use of composite materials eventually seeded with different cell types. However, in the specific pediatric case, the biodegradability of the scaffold is a crucial factor to be guaranteed in order to regenerate the urethra as it has to accompany the natural growth of the patient. Another important issue which has to be further evaluated is the choice of animal model as the majority of them consist of surgically created urethral defects, which might not reflect the anatomical and tissue quality challenges posed by hypospadias repair [3].

## 4. Urinary Bladder Reconstruction

Congenital disorders, such as bladder exstrophy, neurogenic bladder, myelomeningocele, and spina bifida, often result in an anatomically or functionally impaired urinary bladder. Reduced bladder capacity, a high-pressure bladder, impaired compliance, poor voiding, and incontinence are the main features, often leading to progressive upper tract damage. Children with myelomeningocele and neuropathic bladder risk upper urinary tract deterioration in more than 50% of cases if not managed properly [117,118]. In these cases, pharmacotherapy and frequent catheterization are needed to reduce intravesical pressure and maintain renal function, which can in turn cause mechanical damage to the urethra and frequent urinary tract infections. However, retention and incontinence issues significantly affect the patient’s quality of life. If medical therapy and self-drainage are not sufficient, the treatment of neurogenic bladder is surgical: from minimally invasive approaches (e.g., Botox injections and neurostimulator implants) to more invasive interventions when complex surgical reconstruction is needed [51].

Currently, the gold standard therapy is enterocystoplasty, which consists in bladder augmentation, usually using gastrointestinal tissue, to create a reservoir that can store urine at low pressure and help to achieve continence, while protecting the upper urinary tract and consequently preventing the need for dialysis or kidney transplantation. However, this method is often associated with metabolic disturbances caused by absorption of urine by the bowel, infections, stone formation, excessive mucus production, bladder perforation, B_12_ deficiency, bowel obstruction, and even malignances [119]. Moreover, the bowel tissue can be insufficient in some cases.

With the aim of overcoming these drawbacks, the attention has been focused on tissue engineering as a novel treatment approach which could spare healthy bowel tissue. The goal is to guarantee the bladder’s capacity to store urine for prolonged periods while restoring the barrier effect to prevent the passage in the bloodstream of highly permeable molecules eliminated in urine. In normal bladder tissue, specialized cells (umbrella cells) form the permeability barrier as they are interconnected by high-resistance tight junctions, which block transepithelial ion flux. On the other hand, intestinal tissue has the opposite function as it is designed to absorb solutes from the gut lumen into the bloodstream, and this is the main cause of the complications reported in the current clinical procedure.

For these reasons, bladder regeneration with cells derived from the patient’s own tissue may represent an attractive option, particularly in the pediatric population where there is a strong need for living functional tissue with a growth potential and a long lifespan.

Tissue engineering has been explored as an alternative to enterocystoplasty for the management of neurogenic bladder dysfunction by utilizing biodegradable scaffolds, either unseeded or seeded with primary cells, in both animal models [68,120,121] and clinical trials [34,122].

Synthetic materials that have been tested in experimental and clinical settings include polyvinyl sponges, Teflon, collagen matrices, Vycryl (PGA) matrices and silicone; however, most of them failed because of mechanical, structural, functional, and biocompatibility issues.

The first attempts to replace the urinary bladder were performed in the 1950s when Bohne et al. [123] and Portilla Sanchez et al. [124] implanted plastic substitutes as temporary bladder substitutes in patients. In 1957, Bohne et al. [123] used plastic molds for bladder reconstruction in seven patients following subtotal cystectomy. The plastic mold was implanted orthotopically for several weeks and then removed. The regenerated pseudo-bladder was characterized by fibrotic tissue and underwent contraction over time. Moreover, vesicoureteral reflux, dilatation of the upper urinary tract, recurrent urinary tract infections, and the eventual deterioration of renal function occurred. Unluckily, this experiment failed in all patients. Afterwards, in 1958 and 1964, there were other clinical experiments using plastic implants for bladder substitution [124,125], but the complications and the high mortality caused the abandonment of this technique. However, a noteworthy observation is possibly due to these early studies: the urothelium has the capability to migrate and proliferate, while, conversely, smooth muscle regeneration was not achieved in any patient with this method.

Kudish [126] tested the use of a polyvinyl sponge for the replacement of surgically created bladder defects in six dogs. However, this synthetic material was unsuccessful because of a lack of adequate collagen infiltration, wound sepsis, calcium salt deposition, and excessive compression of the material, which caused unfavorable results.

Afterwards, Tsuji et al. [127] and Orikasa et al. [128] tested the first biomaterial used for reconstruction of the urinary bladder: gelatin sponge. This material, treated with an alcohol or a synthetic resin (nobecutane) was used for both augmentation cystoplasty and bladder replacement following subtotal cystectomy. It provided a temporary scaffold for tissue growth and underwent remodeling and degradation over time. However, the unsatisfactory results in terms of smooth muscle regeneration led to the investigation of other types of materials. This technique was re-evaluated by Taguchi et al. [129], who demonstrated that nobecutane-treated gelatin remained in the regenerated bladder wall and caused vesical stones and other undesirable effects. For this reason, the authors opted for Japanese paper, which was expected to be harmless and could be removed completely through the urethra without difficulty after the formation of the granulated tissue. They enlarged the contracted bladders of 13 patients by placing thin paper covered with liquid synthetic resin (nobecutane) like a cap over the opened bladder tissue. Within 3–4 weeks, granulation tissue completely covered the artificial cap, which was removed transurethrally. Excellent results were obtained in 11 patients with tuberculous contracted bladders, and the patients regained normal bladder capacity and micturition, while in 2 patients with interstitial cystitis this technique was not effective. 

Kelami et al. [130] performed investigations of bladder regeneration using Teflon-felt as a bladder wall substitute in 52 dogs, showing how regeneration occurred only in the uroepithelium and not in the musculature. The authors concluded that Teflon-felt could be used as a patch to cover bladder wall defects and that it could be an excellent material for the permanent closure of urinary fistulas.

More recently, other biomaterials have been investigated, including collagen biomatrix [131], bovine pericardium [132], and dura [133].

Lyophilized human dura was evaluated by Kelami et al. [134,135] and Arikan et al. [133] for bladder reconstruction. Kelami et al. [134] tested cystoplasty by using dura mater (6 × 14 cm^2^) in 34 patients (6 with bladder resection-contracted bladders and 28 with bladder wall resection due to bladder carcinoma). After 10–12 weeks, the dura was completely absorbed, and the follow-up lasted 2–6 years. There were no differences in appearance between the regenerated and the native epithelium. The reconstructed bladders were well vascularized, and there were no signs of contractions. However, there were no signs of muscle regeneration. Afterwards, Kelami et al. [133] used dura (6 × 14 cm^2^) for augmentation cystoplasty in neurogenic bladder dysfunction (seven patients with spinal trauma and three with myelomeningocele). The cystometric capacity and intravesical pressure were improved, and normal transitional epithelium was revealed. However, again only weak smooth muscle regeneration was observed.

On the other hand, Moon et al. [132] reported a case of enterovesical fistula repair using bovine pericardium (2.4 × 2 cm^2^). However, the results were questioned because of the small area and the lack of data regarding bladder capacity and compliance.

It was soon clear that non-biodegradable synthetic scaffolds used for bladder reconstruction are usually prone to mechanical failure and urinary stone formation, while biodegradable ones can lead to fibroblast deposition, scarring, graft contracture, and reduced reservoir volume over time, especially in a non-seeded configuration. Consequently, studies were then mainly focused on biodegradable scaffolds for urinary bladder reconstruction, eventually involving the use of seeded cells in order to enhance tissue regeneration.

The first short-term clinical trial related to bladder engineering was performed by Atala et al. [34], who treated seven patients with myelomeningocele (4–19 years old) with biodegradable bladder-shaped scaffolds made of homologous decellularized bladder submucosa (four patients) and composite scaffolds made of collagen and PGA (three patients), seeded with autologous UCs and SMCs. The composite scaffold of collagen and PGA seeded and wrapped in omentum to support vascularity showed promising results. However, phase II studies at 3 years post-implantation failed to show significant improvements in bladder capacity or compliance within the neurogenic bladder patients [122]. In this study, 11 patients (3–16 years old) with neurogenic bladder due to spina bifida received a PGA/PLA biodegradable scaffold (Tengion) seeded with autologous UCs and SMCs. There was no improvement in bladder capacity at 1 and 3 years in any patient. Moreover, adverse events occurred in all patients, including bowel obstruction and bladder rupture.

Another promising biodegradable xenogeneic collagen-based tissue is SIS, which was demonstrated to promote regeneration on a variety of host tissues and which was used in non-seeded configuration for bladder augmentation, demonstrated the ability to regenerate in vivo [23,136]. Kropp et al. [137,138] evaluated its use as a possible bladder augmentation material in the case of rats and dogs. They determined the feasibility of promoting urinary bladder regeneration with porcine SIS in 22 rats which underwent partial radical cystectomy with immediate bladder augmentation [138]. At 48 weeks, all three normal bladder layers (urothelium, smooth muscle, and serosa) were present. Soon after, they evaluated the use of SIS as a possible scaffold for bladder augmentation in 19 male dogs [23]. All the dogs survived without morbidity and mucosa; smooth muscle and serosa layers of the normal bladder showed evidence of regeneration, suggesting the potential use of SIS as a scaffold for bladder augmentation. However, SIS-based grafts (Surgisis and Integra), used for augmentation cystoplasty in lambs, were associated with fibrosis by Kumar et al. [139]. Better, but not sufficiently satisfying, results were obtained by Caione et al. [131], who used SIS in five patients (three males and two females with a mean age of 10.4 years), presenting poor bladder capacity and compliance after complete exstrophy repair. Bladder regeneration was feasible in these patients; however bladder capacity and compliance were too poorly increased to obtain significant clinical benefits. Moreover, poor muscle components were observed in comparison with native tissue.

Subsequently, the use of SIS (Surgisis) was tested in eight patients with poor bladder capacity and compliance (age from 14 to 54 years old), leading to an increase in bladder compliance and capacity and a decrease in maximum detrusor pressure, with no metabolic consequences or urinary calculi formation [140].

Studies were also focused on the comparison of SIS with other types of scaffolds. Portis et al. [141] performed bladder augmentation using laparoscopic techniques on minipigs, using porcine bowel acellular tissue matrix (ATM), bovine pericardium (BPC), human placental membranes (HPM), and porcine SIS. At 12 weeks, the grafts had contracted post-operatively to 70%, 65%, and 60% of their original sizes, respectively, and only mucosal regeneration was demonstrated. The long-term results at 1 year were evaluated by Landman et al. [142], who demonstrated muscle formation at the SIS graft periphery and center, but it consisted of small fused bundles with significant fibrosis. Nerves were also present at the graft periphery and center, but they were decreased in number. They concluded that no advantages in bladder capacity and compliance could be demonstrated.

Moreover, the use of seeded cells in combination with SIS was investigated. Chung et al. [143] compared a control group (sham operation) with partial cystectomy with an oversewn defect group (OG), augmentation with an unseeded SIS group (USG), and augmentation with a seeded SIS group (SSG), with stem cells derived from bone marrow, in 33 rats. The authors noted better results in terms of bladder reconstitution in the case of SSG.

Similarly, Sharma et al. [144] used bone marrow MSC-seeded SIS to perform augmentation cystoplasty in primates, showing urothelial and smooth muscle growth, but no increase in bladder volume (with a recovery between 28% and 40% of native bladder capacity).

More recently, Frimberger et al. [145] evaluated the capability of a human embryonic germ (hEG) cell-derived cell line (SDEC), seeded on porcine SIS, to regenerate the injured rat bladder (30 rats tested, 15 with unseeded SIS and 15 seeded). No graft rejection or diminution in bladder capacity occurred. Unlike with the unseeded SIS, no calcareous deposits were observed. The rat bladder was completely regenerated 28 days after cell-seeded SIS implantation.

In addition to SIS, another promising material evaluated for bladder reconstruction was BAM. Yoo et al. [146] used allogenic bladder submucosa obtained from dogs for augmentation cystoplasty in 10 beagle dogs (5 implanted with unseeded graft and 5 implanted with seeded graft with UCs on one side and SMCs on the other side). The bladders augmented with seeded scaffolds showed a 99% increase in capacity compared with the unseeded ones, which demonstrated a 30% increase in capacity. All the dogs showed a normal bladder compliance and contained a normal cellular organization consisting of a urothelial lined lumen surrounded by submucosal tissue and smooth muscle. 

Obara et al. [120] assessed the feasibility of BAM in spinal cord-injury rats. The regeneration of urothelium, smooth muscles, and nerve fibers was demonstrated in the grafted BAM, which also showed the proper storage function.

Urakami et al. [121] studied bladder augmentation by using BAM to improve the function of spinal cord injury (SCI)-mediated neurogenic bladder in 35 female rats. Seventy-one percent of the rats developed hyperreflexic bladders, and 29% had underactive bladders before bladder augmentation. An improvement was demonstrated in some bladder functions in both the hyperreflexic and the underactive bladders after augmentation. Bladder compliance was increased in the hyperreflexic bladders and decreased in the underactive bladders. Bladder augmentation decreased bladder capacity in high-capacity rats and increased it in low-capacity rats. After 8 weeks, a complete regeneration of BAM, including neovascularity, smooth muscle, and urothelium regeneration and re-innervation was observed, and the voiding function in SCI-induced neurogenic bladders was improved.

Additionally, some authors tested the use of specific growth factors in order to enhance tissue regeneration. Kikuno et al. [68] evaluated the combined use of nerve growth factor (NGF) and vascular growth factor (VEGF) on the regeneration of BAM in SCI-mediated neurogenic bladder in 40 female rats. The rats were divided into 5 groups: group 1 received only spinalization surgery, group 2 received BAM with no growth factors at 8 weeks after the spinalization surgery, group 3 and 4 received NGF or VEGF respectively, and group 5 received both growth factors. The authors concluded that NGF had a significant synergistic effect on the development, differentiation, and functional restoration of BAM when administered with VEGF in neurogenic bladder.

Instead, Zhou et al. [147] tested the use of platelet-derived growth factor-BB (PDGF-BB) and vascular endothelial growth factor (VEGF) in a BAM scaffold (experimental group) in comparison with BAM alone (control group) for bladder augmentation in a rabbit model. The experimental group showed better strip contractility, smooth muscle regeneration, and vascularization in comparison with the control group, while no differences were found regarding urothelium.

In addition to the use of growth factors, other seeded cell types were also tested with the aim of promoting regeneration. For example, Zhe et al. [148] demonstrated muscle cell migration in the case of BAM seeded with adipose-derived stem cells (ASCs), followed by intraperitoneal incubation for bladder reconstruction in a rat model of bladder augmentation. Moreover, a greater bladder capacity was observed in comparison with the unseeded group and significantly more nerve cells.

In another study, the use of a whole decellularized urinary bladder in 16 rabbits (8 with scaffold not in direct contact with urine and 8 with scaffold in direct contact with urine) was tested [149]. The authors demonstrated the absence of fibrosis and inflammatory changes in the first group, in contrast to the second group where a significantly higher grade of fibrosis was observed.

With the aim of facilitating the growth of urothelial and smooth muscle cells, SF has also been evaluated as a scaffold for bladder reconstruction. Zhao et al. [150] developed a bi-layered scaffold comprising SF and BAM and evaluated its feasibility and potential for bladder regeneration in a rat bladder augmentation model. The composite scaffold promoted smooth muscle, blood vessels, and nerve regeneration in a time-dependent manner. After 12 weeks, composite scaffolds displayed superior structural and functional properties without significant local tissue responses or systemic toxicity. Moreover, multilayer urothelium, an extracellular matrix-rich lamina propria, and an outer layer of smooth muscle bundles that resembled the tissue architecture of native control tissue were observed. However, bladder stones, graft perforation, and chronic inflammatory response were observed.

Bi-layer SF and SIS demonstrated the promotion of defect consolidation and mediated functional voiding in non-diseased animal models of bladder augmentation [23,137,151,152,153,154]. 

Several other types of materials were investigated for bladder reconstruction. Mauney et al. [151] studied the efficacy of a gel spun silk-based matrix for bladder augmentation in a murine model. After 70 days from implantation, the silk matrices could support both UC and SMC regeneration. Moreover, the gel spun silk matrices elicited a minimal acute inflammatory reaction, in contrast to the parallel assessments of SIS and PGA matrices, which promoted evidence of fibrosis and chronic inflammatory responses. Silk augmented mice displayed a similar voiding pattern in comparison to the non-surgical controls and supported significant increases in bladder capacity, voided volume, and flow rate while maintaining similar compliance relative to the control group.

Gomez et al. [152] determined the impact of the winding and post-winding fabrication parameters of multi-laminate SF on the in vivo performance in a murine model of bladder augmentation. Three silk matrix groups with distinct structural and mechanical properties were studied after 10 weeks from implantation. The authors demonstrated how alterations in the fabrication parameters can enhance the degradation rate of gel spun silk scaffolds in vivo while preserving the ability to support bladder regeneration and function.

Seth et al. [153] compared three distinct groups of 3D matrices obtained from SF (group 1 and 2 by a gel spinning technique and group 3 by a solvent-casting/salt-leaching method in combination with silk film casting), which were put in comparison with SIS. The SF groups and SIS were evaluated in a rat model of augmentation cystoplasty after 10 weeks of implantation, showing how the variations in scaffold processing techniques can influence the in vivo functional performance of SF in bladder reconstruction. The animals belonging to group 3 displayed superior urodynamic characteristics, including compliance, functional capacity, and spontaneous non-voiding contractions consistent with the control levels. However, there was a high incidence of foreign body reaction in either the silk matrices or the SIS combined with SF.

Tu et al. [154] analyzed two bi-layer matrix configurations of SF (6 × 6 cm^2^) in juvenile swine for 3 months after implantation for bladder augmentation. Next to high rates of survival, voluntary voiding was confirmed over the course of the study period and an increase in bladder capacity and compliance in comparison to the controls. Both matrices supported tissue formation in terms of urothelium and smooth muscle regeneration. Moreover, innervation and vascularization were evident in all the regenerated groups. However, urine leakage, urine calculi, and graft contraction were observed.

Chung et al. [155] investigated the use of bi-layer SF and SIS for bladder regeneration in a rat model of spinal cord injury (SCI). Forty-two female rats were divided into four groups: group 1 (SCI-SIS) was subjected to augmentation cystoplasty with SIS, group 2 (SCI-SF) with bi-layer SF, group 3 (SCI-control) with SCI did not receive a matrix, and group 4 (NS-control) comprised normal rats that did not receive bladder implants. Both implants supported the formation of smooth muscle layers with contractile protein expression as well as the maturation of multi-layer urothelium. Moreover, improvements in certain urodynamic parameters in SCI animals, such as a decreased peak in intravesical pressure following SIS and bi-layer SF implantations, were observed. Both scaffolds were able to support the formation of innervated, vascularized smooth muscle, and urothelial tissues in a neurogenic model. However, bladder stones, bladder rupture, chronic inflammation, and residual silk were present.

In another study [156], the use of acellular dermal biomatrix (AlloDerm, 4 × 4 cm^2^) in obstructed bladder diseased pigs for bladder augmentation was reported. Interestingly, the authors reported unfavorable results in contrast with previous results in healthy animals. In fact, the histological evaluations revealed extensive fibrosis with poorly organized muscle and intense inflammatory cell response. Some degree of shrinkage was noted in repopulated graft segments, which the authors suggested could have been caused by the increasing fibrosis or the remodeling of the matrix in diseased bladders. Blood vessels were disorganized through the entire thickness of the graft.

In another study, Roelofs et al. [157] used a highly porous bovine type I collagen scaffold (32 mm diameter) in 12 sheep in healthy and diseased models (bladder exstrophy was surgically created at 79 gestation days). The regeneration of the bladder was comparable to the regeneration in the healthy bladder and resulted in tissue of good quality.

Additionally, Vardar et al. [158] developed a multi-layered scaffold consisting of a bioactive fibrin layer laminated between two collagen sheets obtained from rat tails, all having undergone plastic compression, in order to perform bladder augmentation in a rat model after partial bladder excision. Moreover, the fibrin was functionalized with a recombinant human insulin-like growth factor-1 (IGF-1) variant, which triggered host SMC invasion. 

Finally, Leonhäuser et al. [159] tested unseeded and seeded collagen scaffolds (OptiMaix 2D and 3D) with autologous UCs and SMCs in six minipigs which underwent cystoplasty surgery. Both scaffolds had a good ingrowth capacity in vivo into the bladder wall, including a quick lining with UCs. The ingrowth of the detrusor muscle tissue, along with the degradation of the scaffolds was also observed. Moreover, Winde et al. [160] tested a biocompatible collagen mesh (Lyoplant) derived from acellular bovine pericardium in a bladder defect rat model for bladder augmentation. After 5 weeks, the implants showed an adequate incorporation, significant cell infiltration, and neovascularization.

To date, several types of scaffolds and cells have been evaluated in order to reconstruct the urinary bladder, but various animal models and surgical repairs have also been investigated (in Table 3 and Table 4, the studies performed in animal models and in patients are summarized, respectively). However, scientists have not yet come across the ideal solution, even though biodegradability seems to be a crucial feature, especially in the pediatric field. Additionally, if urothelial regeneration can be more easily obtained, muscle, nerve and, vascular regeneration cannot be achieved without the pre-seeding of a scaffold, eventually combining the use of specific growth factors.

## 5. Conclusions

Congenital malformations such as hypospadias and neurogenic bladder are complex diseases whose management still remains challenging despite the innovations and refinements of the surgical techniques. The limited supply of usable autologous functional tissues has led to the need to develop suitable biomaterials for urethral and bladder replacement. 

Seeded biomaterials, such as collagen, keratin, alginate, acellular tissue matrices, and synthetic polymers, generally gave better results in comparison with Teflon, silicone, Vicryl, and polyvinyl in terms of biocompatibility, degradation, cell adhesion, and mechanical properties.

In particular, synthetic materials have been associated with unsuccessful results related to the lack of increasing capacity due to the stiffness of the material and the lack of smooth muscle cell growth, as well foreign body reactions, fibrosis, and incomplete urothelial growth.

On the other hand, biomaterials such as BAM have been successfully used in animal trials because of their good biocompatibility. However, acellular grafts did not demonstrate the promotion of the ingrowth of smooth muscle cells. 

Despite the promising results of SF in terms of the growth of urothelial and smooth muscle cells, due to the high risk of inflammation, stone production, leakage, and the persistence of SF remnants, this particular material has to be further investigated for bladder augmentation.

In the light of the several studies performed, it became clear how imperative it is to quickly reconstitute urothelium in order to prevent urine leakage and the development of peritonitis. Luckily, in many studies the urothelial layer formation was more easily obtainable than smooth muscle one, which is just as imperative to obtain, especially in the case of the urinary bladder, whose functionality is regulated by SMCs. In fact, reconstruction of the urinary bladder requires smooth muscle regeneration because its function mainly depends on the compliance and contractility of the detrusor muscle. 

Regarding cells, the best results were achieved in the case of seeded scaffolds with autologous UCs and SMCs as urothelium regenerates spontaneously, while the smooth muscle compartment heals via repair through scar formation.

However, in some cases, the use of autologous urinary cells is not an option due to their limited quality and functionality and, in the case of malignancy, their risk of promoting relapse. For these reasons, other cells sources have been investigated in order to obtain both differentiated UCs and SMCs, for example by starting from MSCs from bone marrow or adipose tissue.

Moreover, the combined use of growth factors (already present in acellular natural derived scaffolds) could promote vascularization, smooth muscle cell repair, and ingrowth and functionality, such as in the case of IGF-1, PDGF-BB, and VEGF.

In the specific case of bladder engineering, the ultimate goal consists in the construction of a complete, physiologically functional bladder. However, as a first objective, even the construction of a passive, catheterizable reservoir for urine, utilizing tissue engineering instead of deploying ileal segments, would carry significant benefits. From this perspective, bladder innervation could be a secondary necessity. 

Moreover, an important issue to face is not only the choice of animal model for urethral reconstruction and bladder augmentation, but also the choice of disease model instead of a healthy one. In fact, biomaterials such as SIS and BAM showed excellent tissue regeneration in healthy animal models with the contractility of a muscle component; however, controversial results were obtained in some cases of the diseased animal models. In fact, in the case of healthy bladders, compensative expansion of the native bladder can occur even when the growth of regenerated bladder tissue is not sufficient.

Consequently, it became clear that scaffolds working well in healthy urinary tissues could not necessarily be as effective in a diseased model in which the cells that would populate the graft were generally abnormal.

Therefore, the main measure of success for the scaffold should be to demonstrate its ability to improve the capacity and compliance of the bladder and not only the demonstration of tissue layer regeneration.

Minimizing the effects of congenital malformations of the urinary tract remains a challenge for pediatric surgeons. New techniques in scaffolds and cell choice for urethral reconstruction and bladder augmentation could give new future perspectives in terms of reducing side effects and maximizing the quality of life of pediatric patients. Despite the great strides made in urological tissue engineering, several issues still have to be faced in order to improve the results in terms of muscle regeneration, limitation of complications, and functional restoration of urethra and urinary bladder, especially in the case of pediatric patients.

## Figures and Tables

**Table 1:** In vivo tests in animal models for urethral reconstruction.

Scaffold Type	Unseeded/Seeded	Animal Model	Tissue Regeneration	Reference
BAM	Unseeded	Rabbit	Urothelium and smooth muscle regeneration and neovascularization.	[81]
Bladder submucosa	Unseeded + seeded with foreskin epidermal cells	Rabbit	Single layer of epidermal cells with disorganized muscle fibers in unseeded group. Several layers of epidermal cells with abundant vessels in seeded group.	[82]
BAM	Unseeded + seeded with oral keratinocytes	Rabbit	No one-layer or stratified epithelium cells in unseeded group. Multiple layers of keratinocytes in seeded group.	[83]
BAMH/SF	Pre-implantation in omentum	Rabbit	Epithelium and smooth muscle regeneration.	[84]
PABM vs. dermis	Unseeded	Pig	PABM was extensively vascularized and completely infiltrated by cells, while dermis remained acellular.	[95]
Amniotic membrane	Unseeded	Rabbit	Complete re-epithelialization. One case of infection and fistula and two cases of urethral strictures.	[96]
AM and BM	Unseeded	Rabbit	Better results with combined AM and BM in terms of neovascularization and epithelial formation.	[97]
PAM	Unseeded	Rabbit	Complete transitional cell layer formation.	[98]
Urethra	Pre-implantation in omentum vs. seeding of MSCs obtained from preputial tissue	Rat	In vivo recellularization provided angiogenesis and cell seeding of epithelium-like cells and SMCs while in vitro recellularization was less effective.	[99]
Collagen scaffold	Unseeded and seeded with epithelial cells and SMCs	Rabbit	Better results in seeded group with normal urethral architecture, maintenance of a wide urethral caliber without strictures.	[101]
2-layer acellular high density collagen tube	Unseeded	Rabbit	UC and SMC repopulation with 20% of both fistula and stenosis.	[12]
2-layer collagen graft	Seeded with oral cells (epithelial and muscle cells)	Dog	Seeded group did not have strictures, leakage or dilatation, while fistula and severe strictures occurred in unseeded group.	[103]
SF vs. SIS	Unseeded	Rabbit	Both scaffolds promoted SMCs and epithelial tissue regeneration. De novo innervation and vascularization were also evident. SIS promoted chronic inflammatory response.	[20]
Composite scaffold made of keratin, silk, gelatin, and calcium peroxide	Unseeded	Dog	Improved organized muscle bundles and epithelial layer.	[104]
TEBM	Seeded with keratinocytes and fibroblasts	Dog	No signs of strictures. Epithelial cells formed stratified layers.	[19]
PTFE	Unseeded	Dog	No evidence of regeneration of normal urethral tissue. Formation of calcification and fistula.	[106]
Polyglactin fiber mesh tube	Unseeded	Dog	Almost complete urethral epithelium regeneration was achieved and neourethral remained patent, no strictures or inflammatory reactions.	[108]
PLLA/PEG	Seeded with amniotic mesenchymal cells	Rabbit	Better results were achieved with seeded scaffolds in terms of smooth muscle and fibrous tissue formation.	[111]

**Table 2:** Urethral reconstruction in patients.

Scaffold Type	Unseeded/Seeded	Patients’ Age	Results	Reference
Collagen matrix obtained from bladder submucosa	Unseeded	4–20 years	Successful in 3 of 4 patients	[47]
Collagen matrix obtained from bladder submucosa	Unseeded	22–61 years	Successful in 24 of 28 patients	[79]
BAM	Unseeded	21–59 years	The success rate was 89% in the case of patients with only one or no previous interventions. The success rate was 33.3% in the case of patients with two or more previous operations.	[80]
SIS	Unseeded	3–18 years	Seventy percent of patients were completely dry (85% in females and 43% in males).	[116]
SIS	Unseeded	61–68 years	Unsuccessful, perhaps due to non-acellularity of SIS scaffold	[87]
SIS	Unseeded	20–74 years	85% success	[89]
SIS	Unseeded	45–73 years	80% success	[90]
SIS	Unseeded	1.5–15 years	Nine of twelve patients voided normally. Six patients had no further interventions and three had small fistulae. In three patients the graft failed.	[91]
Acellular dermal matrix	Seeded with bladder urothelium cells	14–44 months	All patients could void normally. Only one patient developed stricture. Two patients developed a fistula, and one developed an obstruction.	[93]
Acellular dermis matrix	Seeded with UCs	6–8 years	Urinary flow curves were bell-shaped in all but one. Patients with severe hypospadias have high complication rates.	[94]
PTFE	Unseeded	1.5–14 years	One patient developed fistula and all patients developed mild stenosis.	[107]
PGA:PLGA	Seeded with UCs and SMCs	10–14 years	Scaffolds remained functional without fistula and urinary infections.	[112]

**Table 3:** In vivo tests in animal models for urinary bladder reconstruction.

Scaffold Type	Unseeded/Seeded	Animal Model	Tissue Regeneration	Reference
Polyvinyl sponge	Unseeded	dog	The sponge failed to incorporate with normal bladder tissue by firm fibrous union.	[126]
Teflon	Unseeded	dog	Regeneration of urothelium and not muscle.	[130]
BAM	Unseeded	rat	Regeneration of urothelium, blood vessels, smooth muscle, and nerves. Significantly increased bladder capacity and compliance in group injected with NGF and VEGF.	[68]
BAM	Unseeded	rat	Regeneration of urothelium, smooth muscles, and nerve fibers.	[120]
BAM	Unseeded	rat	Regeneration of urothelium, smooth muscle and nerves, and improvement of voiding function.	[121]
BAM	Seeded with ASCs	rat	Greater bladder capacity compared with unseeded group and significantly more nerve cells.	[148]
BAM	Unseeded + PDGF-BB and VEGF	rabbit	Better contractility, smooth muscle regeneration, and vascularization in comparison with control group with no growth factors.	[147]
Bladder submucosa	Seeded with UCs and SMCs	dog	Increase in bladder capacity and normal cell organization of urothelium and smooth muscle.	[146]
Decellularized urinary bladder	Unseeded	rabbit	No fibrosis and inflammatory changes when scaffolds were not in direct contact with urine.	[149]
SF + BAM	Unseeded	rat	Urothelium, smooth muscle, blood vessel, and nerve regeneration.	[150]
SF	Unseeded	mouse	Urothelium and smooth muscle regeneration.	[152]
SF	Unseeded	pig	Urothelium and smooth muscle regeneration.	[154]
SF and SIS	Unseeded	rat	Urothelium and smooth muscle regeneration. Support of innervation and vascularization.	[155]
SIS vs. BAM	Unseeded	rat	Urothelium and smooth muscle regeneration. De novo innervation and vascularization.	[153]
ATM, bovine pericardium, placental membrane, SIS	Unseeded	minipig	Multilayer transitional epithelium in the central portion of SIS. Partial flattened epithelium in ATM graft. No epithelium was found associated with the placental graft, although a few wisps of lamina propria smooth muscle were detected.	[141]
SIS	Unseeded	pig	Small muscle bundles with significant fibrosis. No improvement in bladder capacity and compliance.	[142]
SIS	Seeded with human embryonic cells	rat	Complete regeneration.	[145]
SIS	Unseeded	rat	Regeneration of urothelium and smooth muscle.	[138]
SIS	Unseeded and seeded with stem cells derived from bone marrow	rat	Urothelium, smooth muscle, and nerve regeneration.	[143]
SIS	Unseeded	dog	Regeneration of mucosa, smooth muscle, and serosa layers.	[138]
SIS	Unseeded	lamb	Neovascularization.	[139]
SIS	Seeded with bone marrow MSCs	primate	Smooth muscle regeneration.	
Gel spun silk-based matrix	Unseeded	mouse	Urothelium and smooth muscle regeneration.	[151]
Acellular dermal biomatrix	Unseeded	Pig	Fibrosis and disorganized blood vessels.	[156]
Type I collagen scaffold	Unseeded	sheep	Normal tissue regeneration.	[157]
Fibrin layer laminated between two collagen sheets	Unseeded + IGF-1	rat	Smooth muscle regeneration.	[158]
Collagen scaffold	Seeded with UCs and SMCs	minipig	Urothelium and muscle regeneration.	[159]

**Table 4:** Urinary bladder reconstruction in patients.

Scaffold Type	Unseeded/Seeded	Patients’ Age	Results	Reference
Plastic mold	Unseeded	38–64 years	No tissue regeneration. Contracted bladder.	[123]
Plastic mold	Unseeded	65 years	Complete urothelium formation, no muscle formation.	[124]
Plastic mold	Unseeded	Not reported	Complete urothelium formation, no muscle formation.	[125]
Gelatin sponge	Unseeded	Not reported	Decrease in bladder capacity, urinary incontinence, vesicoureteral reflux.	[127]
Gelatin sponge + nobecutane	Unseeded	15–46 years	Hydroureteronephrosis urinary leakage.	[128]
Japanese paper + nobecutane	Unseeded	19–52 years	Regeneration of urothelium and muscle. Normal bladder capacity and micturition in patients with tuberculous contracted bladders and not effective in patients with interstitial cystitis.	[129]
Lyophilized dura	Unseeded	9–51 years	Complete regeneration of urothelium, but week smooth muscle. No complications.	[133]
Bovine pericardium	Unseeded	67 years	No complications.	[132]
SIS	Unseeded	8–17 years	Complete urothelium regeneration and partial smooth muscle regeneration. No complications.	[131]
Decellularized bladder submucosa and composite scaffold made of collagen and PGA	Seeded with UCs and SMCs and wrapped in omentum	4–19 years	Urothelium and smooth muscle regeneration. Decrease in leaking point pressure and increasing of volume and compliance. No calculi.	[34]
PGA/PLA	Seeded with UCs and SMCs	3–16 years	No improvement in bladder capacity. Bladder rupture occurred.	[122]

## Data Availability

Not applicable.

## Abbreviations

AM	amniotic membrane
ASC	adipose-derived stem cells
ATM	acellular tissue matrix
BAM	bladder acellular matrix
BM	buccal mucosa
BPC	bovine pericardium
CPO	calcium peroxide
ECs	epithelial cells
hAMSCs	human amniotic mesenchymal cells
HPM	human placental membranes
IGF-1	insulin-like growth factor-1
NGF	nerve growth factor
PABM	porcine bladder acellular matrix
PAM	preputial acellular matrix
PCL	Polycaprolactone
PDGF-BB	platelet-derived growth factor-BB
PEG	poly(ethylene glycol)
PLA	Polylactide
PLCL	poly-L-lactide-co-ε-caprolactone
PLGA	poly(lactic-co-glycolic acid)
PLLA	poly(L-lactide)
PTFE:	Polytetrafluoroethylene
SCI	spinal cord injury
SF	silk fibroin
SIS	small intestinal submucosa
SMCs	smooth muscle cells
TE	tissue engineering
TEBM	tissue-engineered buccal mucosa
UCs	urothelial cells
VEGF	vascular growth factor
